# Supplementary material for: The molecular basis for an allosteric inhibition of K+-flux gating in K2P channels
Source: eLife. 2019 Feb 26;8:e39476. doi: 10.7554/eLife.39476 (PMC6391080; doi:10.7554/eLife.39476)
Supplement: Supplementary file 1. [file elife-39476-supp1.pptx]

## Slide 1
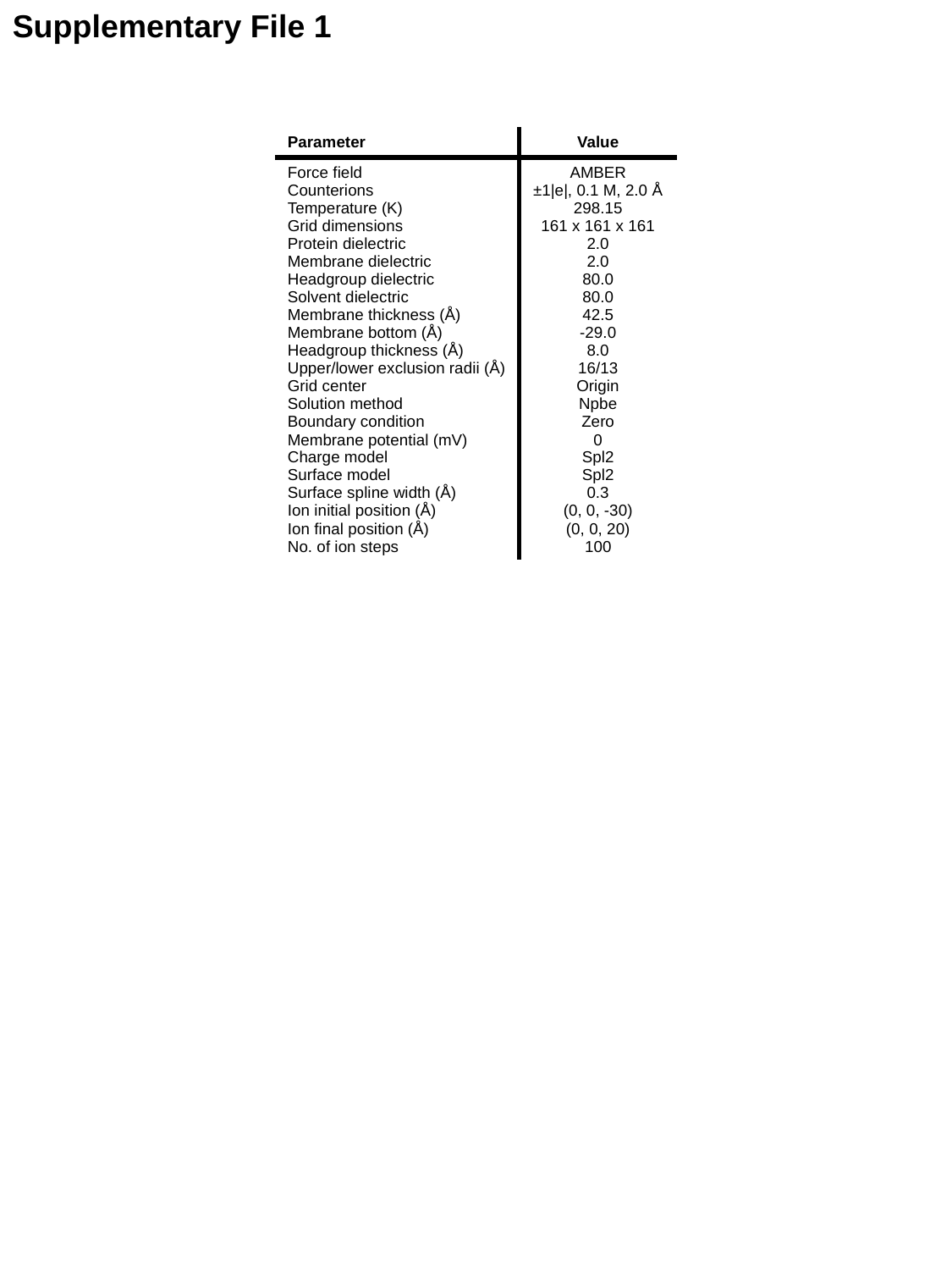

Supplementary File 1
| Parameter | Value |
| --- | --- |
| Force field Counterions Temperature (K) Grid dimensions Protein dielectric Membrane dielectric Headgroup dielectric Solvent dielectric Membrane thickness (Å) Membrane bottom (Å) Headgroup thickness (Å) Upper/lower exclusion radii (Å) Grid center Solution method Boundary condition Membrane potential (mV) Charge model Surface model Surface spline width (Å) Ion initial position (Å) Ion final position (Å) No. of ion steps | AMBER ±1|e|, 0.1 M, 2.0 Å 298.15 161 x 161 x 161 2.0 2.0 80.0 80.0 42.5 -29.0 8.0 16/13 Origin Npbe Zero 0 Spl2 Spl2 0.3 (0, 0, -30) (0, 0, 20) 100 |
